# The prevention and reduction of weight loss in an acute tertiary care setting: protocol for a pragmatic stepped wedge randomised cluster trial (the PRoWL project)

**DOI:** 10.1186/1472-6963-13-299

**Published:** 2013-08-08

**Authors:** Alison L Kitson, Timothy J Schultz, Leslye Long, Alison Shanks, Rick Wiechula, Ian Chapman, Stijn Soenen

**Affiliations:** 1 School of Nursing and Centre for Evidence-Based Practice SA (CEBSA), University of Adelaide, Adelaide, SA 5005, Australia; 2 Green Templeton College, University of Oxford, Oxford, UK; 3 Australian Patient Safety Foundation, University of South Australia, Mailbox CEA-20, Adelaide, SA 5000, Australia; 4 Clinical Dietetics, Royal Adelaide Hospital, North Terrace, Adelaide, SA 5000, Australia; 5 Centre of Research Excellence (CRE) in Translating Nutritional Science to Good Health, School of Medicine, University of Adelaide, Adelaide, SA 5005, Australia

**Keywords:** Malnutrition, Stepped wedge, Nutritional decline, Nutritional screening, MUST, Nutritional support, Oral nutritional supplement, Red tray, Evidence implementation, Knowledge translation, Feeding assistance, Nursing

## Abstract

**Background:**

Malnutrition, with accompanying weight loss, is an unnecessary risk in hospitalised persons and often remains poorly recognised and managed. The study aims to evaluate a hospital-wide multifaceted intervention co-facilitated by clinical nurses and dietitians addressing the nutritional care of patients, particularly those at risk of malnutrition. Using the best available evidence on reducing and preventing unplanned weight loss, the intervention (introducing universal nutritional screening; the provision of oral nutritional supplements; and providing red trays and additional support for patients in need of feeding) will be introduced by local ward teams in a phased way in a large tertiary acute care hospital.

**Methods/Design:**

A pragmatic stepped wedge randomised cluster trial with repeated cross section design will be conducted. The unit of randomisation is the ward, with allocation by a random numbers table. Four groups of wards (n = 6 for three groups, n = 7 for one group) will be randomly allocated to each intervention time point over the trial. Two trained local facilitators (a nurse and dietitian for each group) will introduce the intervention. The primary outcome measure is change in patient’s body weight, secondary patient outcomes are: length of stay, all-cause mortality, discharge destinations, readmission rates and ED presentations. Patient outcomes will be measured on one ward per group, with 20 patients measured per ward per time period by an unblinded researcher. Including baseline, measurements will be conducted at five time periods. Staff perspectives on the context of care will be measured with the Alberta Context Tool.

**Discussion:**

Unplanned and unwanted weight loss in hospital is common. Despite the evidence and growing concern about hospital nutrition there are very few evaluations of system-wide nutritional implementation programs. This project will test the implementation of a nutritional intervention across one hospital system using a staged approach, which will allow sequential rolling out of facilitation and project support. This project is one of the first evidence implementation projects to use the stepped wedge design in acute care and we will therefore be testing the appropriateness of the stepped wedge design to evaluate such interventions.

**Trial registration:**

ACTRN12611000020987

## Background

### Nutritional status of hospitalised patients

The prevalence of malnutrition, defined here as *protein-energy under-nutrition causing measurable adverse effects on tissue/body form and function and clinical outcome*[1] (p. S4), ranges from 20-60% in older patients in Western acute healthcare [1-4]. Additionally, during hospitalisation nutritional status often declines in older patients due to a lack of adequate nutritional intake [5-7]. The adverse clinical outcomes associated with malnutrition and weight loss in acute care include increased: morbidity and mortality, length of stay, rates of infections, pressure sores and readmissions; and decreased functionality [1,8-14]. The economic costs of patient malnutrition and weight loss to healthcare are significant [15,16].

In hospital settings, barriers to preventing nutritional decline have been shown to include a lack of staff knowledge and training [17], lack of prioritisation and timely feeding assistance by nursing staff [18], a lack of coordination between disciplines, including poor interdisciplinary communication, a lack of shared responsibility for nutrition care, and a lack of staff [19]. Naithani, Whelan et al. [20] reported that organisational, environmental and systems issues prevented malnourished patients, or those at risk of malnourishment, from adequately accessing food in the hospital setting. For example, patients were unable to open packaging, missed meals because of scheduled investigatory procedures, were not given food and fluids between mealtimes or these were placed out of reach, and lacked feeding assistance or were interrupted during mealtimes [20].

### Interventions to prevent nutritional decline in hospital patients

The question of how to prevent weight loss and nutritional decline (these terms are used interchangeably in this protocol) in acute patients is complex. Interventions to improve patients’ nutritional status and clinical outcomes have focussed on (i) the use of malnutrition screening tools, (ii) provision of nutritional support including supplements, and (iii) feeding assistance.

Nutritional screening in hospitals is required for accreditation in the US by the Joint Commission (http://www.jointcommission.org), is recommended in the UK by NICE [21] and the British Association for Parenteral and Enteral Nutrition (http://www.bapen.org.uk) and in Australia by the Dietitians Association of Australia as the first step in early detection of at risk patients [1,22]. Typically conducted by nurses [23], the evidence base for improved clinical benefits from screening, and subsequent timely and appropriate referral for nutrition care, is growing [24-26]. However, barriers to implementing nutrition screening tools in clinical practice are commonly encountered; screening rates in hospitals are typically only 60-70% and sometimes much lower [27-31]. A lack of time for what is considered a low priority is commonly cited by nursing staff as a barrier to nutritional screening of patients, additional reasons include lack of training, skills, knowledge and support, patients’ short stays, and a need for enhanced collaboration between dietitians and nurses [27,29,30,32].

Of the large number of available nutritional screening tools [23], the Malnutrition Universal Screening Tool (MUST) developed in 2003 by the British Association for Parenteral and Enteral Nutrition [33] has been widely used [34-37]. The MUST has been shown to be quick (3–5 minutes) and accurate across a variety of hospital patient groups [26,37] and may be completed without weighing patients by using reliable patient or relative memory/recall [26,28]. However, the need for anthropometric measures and the presence of communication difficulties with confused patients are cited as limitations to using MUST [29,30]. Although some studies have claimed it ‘easy’ to use [37], other studies have reported difficulties in using the MUST [30,38].

Nutritional support aims to improve total energy and nutrient intake and may involve provision of oral nutritional supplements (ONS), or, if indicated, enteral tube feeding and/or parenteral nutrition [39]. There are a number of systematic reviews examining the effectiveness of ONS in improving nutritional status and clinical outcomes [5,21,40,41]. Although the review findings are somewhat inconsistent, the evidence suggests that supplementation produces small but consistent weight gain in older people and that improvements in mortality, morbidity (including complication rates) and functional status are greatest in underweight/undernourished patients [5,40,41]. The mean length of stay is not significantly reduced through ONS use [5].

The need for assisted eating is defined as “needing help from another person to be able to eat” ([42], p. 258). Assisted feeding ranges from verbal and non-verbal prompts to physical guiding to transferring food from the plate to an individual’s mouth [43]. Up to 70% of elderly hospital patients require some feeding assistance [44], which is increasingly delivered by patient care assistants as opposed to nurses whose role at mealtimes and responsibilities for patient’s nutritional status has diminished in recent years [18,45,46]. The use of a red tray to identify patients in need of feeding assistance has been advocated across the UK [47-49] and also implemented in a South Australian hospital. Although some studies have shown that nutritional intake can be improved [50,51], the evidence for the effectiveness of feeding assistance in improving patient outcomes is somewhat mixed [43,52,53].

Whilst there is a growing body of scientific evidence for single-faceted nutritional interventions in preventing nutritional decline and, in some cases, improving outcomes of older patients across a discrete sample of hospital wards, there is little research to guide or evaluate the effectiveness of multi-disciplinary, multi-faceted interventions implemented at an organisational level. The study outlined in this protocol aims to redress this imbalance and specifically sets out to address known barriers to implementing evidence about nutrition into clinical practice. Reflecting the focus on improving patient outcomes, the study is titled ‘Prevention and Reduction of Weight Loss in Acute Care (PRoWL)’.

### Specific aims and hypotheses

The study seeks to address four key questions. Firstly, does a multifaceted intervention incorporating a malnutrition screening tool, nutritional supplements and red trays implemented in a hospital improve outcomes for older, at risk patients? Secondly, does the intervention have any impact on staff-related factors (eg receptivity to evidence, staff behaviour and actions) and hospital processes related to nutrition? Thirdly, does the model of facilitated implementation of nutritional evidence support frontline staff to provide evidence-base care? Fourthly, what are the implications for implementing evidence on nutrition within an organisation in terms of successful strategies, sustainability, and study design?

The whole evaluation of the implementation, which will include a stepped wedge design and a full process evaluation of facilitator and staff experiences, will add to existing strategies for implementing nutritional evidence into practice, thereby addressing questions 3 and 4. The following hypotheses that are most pertinent to this study protocol relate to questions 1 and 2 only:

1.1 The multifaceted nutrition intervention will improve outcomes (e.g. prevent nutritional decline, reduce mortality, readmission rates) for older, at risk patients.

2.1 The multifaceted nutrition intervention will improve staff-related outcomes (e.g. receptivity to evidence, staff behaviour).

2.2 The multifaceted nutrition intervention will improve hospital processes related to patient nutrition (e.g. weighing patients, screening patients, documentation).

## Methods/Design

### The intervention

The evidence-based intervention will consist of three linked activities: the introduction and use of the Malnutrition Universal Screening Tool (MUST), the provision of food supplements to patients identified at risk of malnutrition, and the introduction of a system that uses red feeding trays to flag patients requiring full feeding assistance. A multidisciplinary partnership between implementation science researchers (AK, RW, TS), clinicians (AS, LL) and nutrition researchers (SS, IC) was formed to assist in facilitating and evaluating the intervention.

### The implementation

The intervention will be introduced in a staged way across an entire hospital using a stepped wedge design [54,55] described in more detail in the next section. The implementation of the intervention will be facilitated by a nurse paired with a hospital dietitian. The four nurse leads will be identified prior to the project commencing and their wards will be allocated to one of the four groups using stratified randomisation. For three of the four groups an additional five hospital wards will be randomly assigned to the group; the last group will be assigned an additional six wards meaning that a total of 25 wards are allocated (Table 1). Allocation of wards to groups will be conducted (by author TS) by coding each ward and using a random number generator (MS-Excel) to select codes for each groups. The intervention will be implemented over a two month period across all six wards within a group but staggered sequentially every three months between groups. Control sites will experience usual hospital care until the intervention is delivered. The intervention will be carried out over 12 months, commencing in March 2011.

**Table 1:** Representation of stepped wedge trial design showing four groups and five measurement time periods incorporating control (O) and intervention (X) phases

		**Time period**				
		**1**	**2**	**3**	**4**	**5**
Group/Step	1 (six wards)	0	X	X	X	X
	2 (six wards)	0	0	X	X	X
	3 (six wards)	0	0	0	X	X
	4 (seven wards)	0	0	0	0	X

In this trial, 25 hospital wards are randomised to four groups–three groups of six wards and one group of seven wards.

The facilitating nurse-dietitian pairs will be trained in clinical leadership, evidence translation and the intervention (nutritional screening, supplements and the use of red trays prior to the implementation). The structured training available to facilitators will include: a short fellowship at the Joanna Briggs Institute (http://www.joannabriggs.edu.au) to develop evidence implementation and clinical leadership skills, the use of a tool kit containing materials to support their education and leadership roles, and a training pack of clinical content to deliver education to nurses working in their wards. The training materials will be jointly developed with, and facilitators will be supported by, two members of the research team (AK and RW). The clinical content will be developed prior to the roll out by the facilitators working with a dietitian (AS).

The role of the facilitator pairs will be to identify ‘champions’ on each ward and to work with champions to support the roll out of all three components of the intervention before, during and after the intervention phase. The facilitator pairs will train the champions to deliver education and work with champions to undertake unit level audits to determine local compliance with key nutritional evidence-based clinical processes before and after the intervention is implemented, and will assist champions to feed back these local data at each ward.

### Stepped wedge trial

Though infrequently used and poorly known, awareness and use of the stepped wedge trial design is increasing [54,56-58]. Stepped wedge trials are a type of crossover trial in which different groups (or steps) cross over (ie switch from being a control to a treatment) at different time points [59]. In comparison to other crossover trials, the groups/steps only cross in one direction, from control to treatment. The first time point (Time 1) is a baseline measurement, and groups/steps are then randomised to receive the intervention at subsequent time points (Table 1) until all groups/steps have received the intervention [59]. Data is collected from all groups at regular intervals during the study, including at baseline and whenever a new group receives the intervention. At the conclusion of the trial, each of the units in each group have been exposed to the intervention, and each of the groups has a varying amount of control data collected prior to receiving the intervention (Table 1).

A stepped wedge randomised cluster trial with repeated cross section design will be employed [54,55,59]. The ‘step’ refers to each group of wards; and the ‘cluster’ refers to the ward as unit of randomisation. The ‘repeated cross section’ measures refer to the fact that multiple cross sectional measures are made with different patients in each cluster for each of the sampling time periods [55], as recently used in surgical patients across three hospitals [58].

The stepped wedge allows all clusters to receive the intervention. Further, unlike a conventional crossover trial, no clusters are removed from the intervention, which is ethically preferable if there is the prior belief that the intervention should result in benefits for the patient population [54,55,59]. The design also allows for a staged implementation, which is particularly beneficial if there are logistical, practical or financial limitations to rolling out an intervention at all sites in one time period [59]. The design supports evidence implementation in complex, changeable environments because the staged implementation allows improvements to be made to the intervention, for example training materials can be updated to accommodate feedback from participants. Analysis of stepped wedge trials can adjust for possible temporal trends in the effectiveness of the intervention, and the design reduces the impact of intra-cluster correlations on power because each group acts as its own control [54]. A disadvantage of the design is the need for data collection from all units at each sampling period, and potential lengthening of the trial to allow sequential roll out of the intervention across all groups [54]. These attributes support the use of routinely collected hospital administrative data as outcomes of stepped wedge trials.

### The setting

The study will be conducted in a large tertiary South Australian acute care hospital in which patient care areas are divided into six divisions and a total of 26 wards/units. A recent evidence implementation project on nutrition screening and documentation found substantial improvement from low baseline levels in two hospital wards and identified more widespread interest for further improvement to nutritional status and care of vulnerable patients [60,61]. Additionally, a number of internal, unpublished audits have identified that up to half of patients are at risk of nutritional decline in a system that does not prioritise patient nutrition.

### Inclusion and exclusion criteria

Critical care areas will be excluded from the study, all remaining 25 wards will be allocated to one of the four stepped wedge groups. Otherwise, there are no exclusion criteria for implementing the intervention-all nursing staff and patients on the wards will be eligible to be exposed to the intervention. The patients will all be over 18 years of age with no upper age limitation.

### Outcomes

The primary patient outcome measure will be the rate of change in body mass and body mass index over weekly periods from admission to discharge. Body mass (kg) will be measured to the nearest 0.1 kg, between 0800 and 1200 and post bladder voiding. Depending on patient mobility this may require a standing, sitting, bed or sling weighing machine. Patient’s height (cm) will be measured to the nearest 0.1 cm using a stadiometer or predicted from measured ulna length depending on patient mobility. Body mass index (kg/m^2^) is calculated as body weight divided by height squared. The body mass data will be collected by a researcher (LL), who is not blinded to the randomisation schedule.

Secondary patient outcome measures will include: length of stay, all cause mortality, discharge destination (to a higher, lower or equivalent level of care), number and duration of re-admissions (defined as an unplanned admission within 28 days of discharge related to the primary admission [62] (3, 6, 12 months post-discharge), and number of Emergency Department (ED) presentations (3, 6, 12 months post-discharge). The re-admission and ED presentations data will be collected from across all five major public hospitals in the metropolitan region using the State’s patient record systems, which will also provide length of stay, discharge destination and mortality, cross-referenced with State death registries. The collector of the secondary patient outcome data will be blinded to the randomisation schedule.

The staff-related outcome is nurse perspectives of context of care as assessed by administration of the Alberta Context Tool (ACT) [63,64] before the intervention.

Hospital process-related outcomes include: compliance to using the MUST tool on all patients, compliance to using additional food supplements when indicated, compliance to using the red tray system when indicated and compliance to documenting patient’s foods and fluids intake by nurses. The hospital process measures will be obtained through medical record review.

### Sampling

The primary outcome data will be collected on a sample of patients from only one ward per group due to resource constraints. Given that the median length of stay in the hospital is only 4–5 days, and our requirement is to collect weight data over at least a 1 week period, we will purposively sample a ward that is most likely to care for patients who have a length of stay in excess of 7 days. To further minimise the number of patients for whom a weight on admission is collected but who are discharged before 7 days, patients with Diagnostic Related Groups (DRGs) known to have longer than average length of stay will be targeted. The following patients will be excluded from body mass measurement: those undergoing palliation or expected to die within a month, those undergoing amputation or surgical removal of large tumours, those admitted directly to the intensive care unit or with stays in intensive care of more than 3 weeks. A total of 20 patients for each of four wards will be weighed on each of five measurement periods, resulting in a total sample size of 400. Whilst it was desirable to include either more wards or more patients per ward (see power calculations in Analysis) in the sample, resource constraints limited the final sample size available for analysis. Given the size of the wards (typically 20–24 patients each) it is anticipated that it may take repeated weekly visits over an entire month to reach a total sample of 20 patients with body weight measurements for each sampling period. Patients whose length of stay exceeds 14 days will be weighed at day 14 and day 21 to allow calculation of change in weight over the first, second and third weeks of their admission. The second and third week data will both be analysed separately.

Secondary outcomes and hospital process measures will only be collected from the records of patients included in the primary outcome sample, described above. Written consent to weigh patients will not be sought because it is routine practice and part of usual care in the hospital.

### Analysis

Analysis of stepped wedge designs is challenging [54]. The unidirectional aspect of stepped wedged trials complicates analysis because the treatment effect cannot be estimated exclusively from within-cluster comparisons [59]. Analytical techniques depend on a number of factors including: the existence of a temporal effect on the outcome, equality or inequality in cluster size, and normality of continuous data. Based on a design using equal cluster sizes, and assuming that there will be a temporal effect, linear mixed models (LMM) will be used [65]. If there are no temporal effects, then within-cluster analysis can be used to estimate the treatment effect. Random effects will be used to model correlation between individuals within the same cluster and measure the variance of individuals in a cluster and variance at the cluster-level [59]. Hussey et al. (2007) provide equations and worked examples for calculating both the within-cluster and between-cluster calculations [59].

We conducted power analysis based on a sub-set of body weight data collected at baseline at four hospital wards. In their first week of admission, the prevalence of patients with (i) BMI greater than or equal to 20 who lost 2 or more kg, and (ii) BMI less than 20 who lost 1 kg or more, was measured as 26.7%. Using equations seven and eight [59], a coefficient of variation $\left(\frac{\tau }{\mu }=\frac{0.056}{0.267}\right)$of 0.21, with four groups of 20 patients measured over five time periods totalling 400 patients, a power of 80% would detect a reduction in the prevalence of weight loss from 26.7% to 6.5%, ie a 75% reduction. If a smaller effect size is selected, such as a reduction in prevalence from 26.7% to 16.5% (a 38% reduction), the power is reduced to 29%.

It is also possible to calculate the required sample size for an adequately (80%) powered study. To detect a smaller effect size (a 38% reduction in proportion of patients experiencing weight loss), the total sample size required is 1800 patients, comprising 90 patients in each of four groups measured across the five time periods. Alternatively, although not feasible for reasons defined above, if all 25 wards were included in the sample and 20 patients were included from each ward at each of five time periods, the resulting study comprising a total of 2,500 patients would have a power of 69% to detect a 38% reduction in proportion of patients experiencing weight loss.

All of the outcomes except for the Alberta Context Tool will be analysed according to the stepped wedge design, as these data are all collected at each of the five measurement time periods of the study. The Alberta Context Tool will be compared using an hierarchical (nested) ANOVA to account for variation within, and between, wards. Analysis will be conducted using Stata software.

### Ethics

The study was approved by the University of Adelaide Human Research Ethics and the hospital’s Ethics Committee.

## Discussion

The vulnerability of older adults to nutritional decline may be both a cause, and an effect, of illness [66]. Therefore, preventing nutritional decline is neither possible nor a realistic goal for all hospitalised, older patients and the difficulties in improving patient outcomes in through nutrition interventions are well known [53,67]. Although nutrition has been consistently defined over the last 150 years in the literature and in practice as one of the core nursing fundamentals of care [68], the evidence suggests that multidisciplinary approaches are most likely to prevent nutritional decline [69], particularly as responsibility for nutrition has diffused between multiple disciplines (e.g. nursing, medicine, dietetics and other allied health such as speech pathology and occupational therapy) [6]. Therefore we have proposed an intervention and developed a research team that is both multidisciplinary and team-led. We believe that the interdisciplinary partnerships built into the intervention and study design will lead to a stronger intervention that is more likely to be successful in the short term and sustainable in the long term. Although medical input into the implementation has been provided via the research team, it is unclear how further engagement with doctors around the importance of nutrition in practice will be obtained.

The multifaceted intervention developed for this project is novel, bringing together evidence-based elements for which local support also existed. Additionally, the intervention has sought to address some of the key factors impacting nutritional intake of older patients, particularly those that make it easier for staff to be aware that a patient is at risk of nutritional decline [20]. The use of MUST as the screening tool in this study reflects previous evidence implementation work conducted in this setting [61]. While there is no ‘gold standard’ for malnutrition screening, apart from familiarity, reasons for selecting MUST in this study include that it: allows for screening and establishing nutritional risk, has been widely used, and has good reliability and validity [70].

The implementation designed for this project is based on the PARIHS framework, which proposes that successful evidence implementation is a function of the evidence, the context and the facilitation [71,72]. To best engage with end-users of the evidence (frontline clinical staff) we have built in two levels of facilitation in the implementation (between nurse-dietitian pairs and wards, and between the research team and the nurse-dietitian pairs). Further we have proposed a novel application of the Alberta Context Tool. As part of the intervention, the tool will be used to assess existing context and as the basis for feedback to wards on their perceived strengths and weaknesses.

Our preliminary work towards completing the proposed study has already identified a number of barriers to implementing the intervention into practice. In particular, we have identified the need for each ward to have access to accurate and appropriate scales (e.g. weigh chairs and beds) with which to weigh patients on admission and weekly thereafter, and the difficulties of adding an additional form (the MUST) to the patient record.

We have selected the stepped wedge trial as an innovative approach to a cluster randomised crossover trial [59] subject to the constraints of (i) limited resources to roll out the intervention across the entire hospital, (ii) a belief that the intervention will do more good than harm, therefore should not be withheld from any part of the hospital [57], and (iii) a desire to be able to flexibly modify aspects of the intervention as befitting a realist evaluation [73]. In this instance, the evaluation of a multifaceted intervention that has evidence for its individual components is, in theory, similar to the indicated use of stepped wedge trials where “*interventions likely to do more good than harm, a stepped wedge design may be particularly beneficial in evaluating interventions being implemented in a new setting, where evidence for their effectiveness in the original setting is available, or for patient safety interventions that have undergone careful pre-implementation evaluation to rule out any collateral damage*” [55] (p. 9).

The study design and analysis are, however, complicated and novel. Review of CONSORT statements for reporting of randomised controlled trials (e.g. [74]) indicates that this design includes a number of non-standard elements that impact the design and reporting of the study. For example, the study is a pragmatic trial, in that it seeks to establish the effectiveness of the intervention in normal practice using a flexibly implemented intervention to directly inform policy and “real world choices” [75]. Secondly, the study assesses a nonpharmacologic intervention, with accompanying challenges around the complexity of the intervention, the expertise of care providers and difficulties in blinding [76,77]. Thirdly, the study uses clustering, which has particular implications for design and analysis, blinding, and recruitment of patients within a cluster [78], and study power [59]. Meeting the individual reporting guidance for each of the three non-standard elements to this study (a *pragmatic*, *cluster* randomised trial of *nonpharmaceutical interventions*) represents a substantial effort to be addressed in the final reporting of the study [75,76,78].

The decision to focus on a single facility in this study is a potential limitation but reflects a number of constraints. Firstly, our work assessing context in this facility has shown substantial variation between wards in many dimensions, including leadership, culture and feedback processes [64]. Therefore, we tailored an intervention that involved ward-based champions to address local contextual factors and assist in the collection and feeding back of local audit data collected at the ward level. Secondly, although based on research evidence, the intervention developed for this project was novel and best piloted at a single site before further testing at a health system-wide level (i.e. multiple organisation involvement) is conducted. Lastly, the limited resources available were most appropriately used by concentrating on a single facility. Another limitation to this study, also related to limited resources, is the absence of biochemical markers of metabolic status, such as transthyretin and albumin [79].

Finally, although the project was originally envisaged as accessing routinely collected [54] patient body mass data, initial investigation of this dataset indicated systematic biases and large gaps rendering it unfit for this purpose. Therefore we decided to prospectively collect body mass data on a targeted sub-section of the population. This approach has introduced limitations related to power, as the study will be under-powered to detect anything but large effect sizes, and in generalisability, as we will purposively sample from the patient population and target those with a length of stay likely to be at least 1 week. However, as we are anticipating the results from this study to be preliminary in nature, and to potentially inform a larger study, this was necessary as the first step towards investigating the multifaceted intervention. Further embedding of nutrition screening and weighing of patients will provide a data source for future studies evaluating long term sustainability of the intervention.

## Conclusion

We have outlined a study that we anticipate will contribute to the knowledge base of nutritional care of patients in hospitals and evidence translation across multidisciplinary healthcare. The pragmatic stepped wedge randomised trial offers a novel solution to implementation science in healthcare, particularly when resources are limited, and/or there is a prior belief that the intervention will do more good than harm.

## Abbreviations

ED: Emergency department; ACT: Alberta Context Tool; UK: United Kingdom; US: United States; MUST: Malnutrition Universal Screening Tool; DRGs: Diagnostic Related Groups; BMI: Body Mass Index.

## Competing interests

The authors declare that they have no competing interests.

## Authors’ contributions

AK: conceived the study (with LL), drafted the first versions of the protocol, brought the research team together, led the project development, chaired the research steering group and will support facilitator pairs with RW. TS: conducted literature review, developed and refined the study design and analysis, and will manage the context assessment. LL: conceived the study (with AK), identified appropriate outcomes and assisted in the implementation plan. AS: developed the dietetic component to the intervention and led dietetic and catering aspects to the implementation, identified appropriate study outcomes. RW: developed the training materials with facilitators and will support facilitator pairs with AK. IC: identified appropriate and clinically sensitive outcomes and assisted with study design. SS: identified appropriate and clinically sensitive outcomes and assisted with study design. All authors were members of the project steering group, and contributed to drafts of the protocol. All authors read and approved the final manuscript.

## Pre-publication history

The pre-publication history for this paper can be accessed here:

http://www.biomedcentral.com/1472-6963/13/299/prepub
